# Lnc-GD2H Promotes Proliferation by Forming a Feedback Loop With c-Myc and Enhances Differentiation Through Interacting With NACA to Upregulate Myog in C2C12 Myoblasts

**DOI:** 10.3389/fcell.2021.671857

**Published:** 2021-08-18

**Authors:** Rui Chen, Si Lei, Yanling She, Shanyao Zhou, Huacai Shi, Cheng Li, Ting Jiang

**Affiliations:** ^1^Guangdong Traditional Medical and Sports Injury Rehabilitation Research Institute, Guangdong Second Provincial General Hospital, Guangzhou, China; ^2^Department of Radiology, The Third Affiliated Hospital, Sun Yat-sen University, Guangzhou, China

**Keywords:** c-Myc, differentiation, lnc-GD2H, Myog, NACA, proliferation, C2C12 myoblast, lncRNA

## Abstract

In the present study, the roles of a novel long non-coding RNA (lncRNA), lnc-GD2H, in promoting C2C12 myoblast proliferation and differentiation and muscle regeneration were investigated by quantitative polymerase chain reaction, western blotting, Cell Counting Kit-8, 5-ethynyl-2′-deoxyuridine (EdU), immunofluorescence staining, luciferase reporter, mass spectrometry, pulldown, chromatin immunoprecipitation, RNA immunoprecipitation assay, wound healing assays, and cardiotoxin (CTX)-induced muscle injury assays. It was observed that lnc-GD2H promoted myoblast proliferation as evidenced by the enhancement of the proliferation markers c-Myc, CDK2, CDK4, and CDK6, percentage of EdU-positive cells, and rate of cell survival during C2C12 myoblast proliferation. Additional experiments confirmed that c-Myc bound to the lnc-GD2H promoter and regulated its transcription. lnc-GD2H promoted cell differentiation with enhanced MyHC immunostaining as well as increased expression of the myogenic marker genes myogenin (Myog), Mef2a, and Mef2c during myoblast differentiation. Additional assays indicated that lnc-GD2H interacted with NACA which plays a role of transcriptional regulation in myoblast differentiation, and the enrichment of NACA at the Myog promoter was impaired by lnc-GD2H. Furthermore, inhibition of lnc-GD2H impaired muscle regeneration after CTX-induced injury in mice. lnc-GD2H facilitated the expression of proliferating marker genes and formed a feedback loop with c-Myc during myoblast proliferation. In differentiating myoblasts, lnc-GD2H interacted with NACA to relieve the inhibitory effect of NACA on Myog, facilitating Myog expression to promote differentiation. The results provide evidence for the role of lncRNAs in muscle regeneration and are useful for developing novel therapeutic targets for muscle disorders.

## Introduction

Skeletal muscle accounts for more than 40% of the human body weight and stores approximately 50–75% of human protein in various forms as the largest protein pool (Frontera and Ochala, 2015). Skeletal muscle development/regeneration involves the activation of muscle stem cells, as well as proliferation, differentiation, and muscle fiber fusion of myoblasts (Silva et al., 2019). Myogenic regulatory factors (MRFs) include myoblast determination protein (MyoD), myogenic factor 5 (Myf5), myogenin (Myog), and muscle-specific regulatory factor 4 (MRF4), which mainly regulate the proliferation, differentiation, and fusion of myoblasts. The myocyte enhancer factor 2 (Mef2) family consists of Mef2a, Mef2b, Mef2c, and Mef2d, which regulate the expression of MRFs and modulate the skeletal muscle-specific transcription process involving MRFs (Liu et al., 2020). Among the above-mentioned muscle-specific factors, Myog is a key transcription factor in skeletal muscle growth, development, and regeneration (Hasty et al., 1993; Zhang et al., 2018a). Homozygous Myog-mutant mice survived fetal development but died immediately after birth and exhibited a severe reduction in all skeletal muscle (Hasty et al., 1993).

In addition to muscle-specific regulators, non-muscle-specific transcription factors also participate in muscle regeneration such as the early polypeptide related complexes (nascent polypeptide-associated complex, NAC) (Jafarov et al., 2012) and c-Myc (Luo et al., 2019). NAC is the first cytoplasmic factor binding with newly synthesized peptide chains from the ribosome, and its α subunit (αNAC or NACA) mainly plays a role of transcriptional regulation. NACA could promote erythrocyte differentiation (Lopez et al., 2005). What’s more, NACA can bind to the Myog promoter, and NACA overexpression inhibits Myog transcription and myoblast differentiation (Jafarov et al., 2012), indicating that NACA plays an important role in the regulation of myoblast differentiation. Reportedly, c-Myc promoted myoblast proliferation and muscle fiber hypertrophy in chickens by targeting the cell cycle pathway. In addition, c-Myc can regulate myoblast proliferation and differentiation *via* transcriptional regulation of miRNAs and long non-coding RNAs (lncRNAs) (Luo et al., 2019).

lncRNAs are a class of non-coding RNAs longer than 200 nucleotides in length and with almost no coding ability (Rinn and Chang, 2012). lncRNAs act through different mechanisms, such as regulating the activity of transcription factors, chromatin modification (Hube et al., 2011; Zhou et al., 2015; Sun et al., 2018; Wang et al., 2019), competitive endogenous RNA (Cesana et al., 2011; Zhang et al., 2018b), DNA methylation (Lijun et al., 2015), and the coding of small peptides (Bi et al., 2017; Matsumoto et al., 2017). Not only are lncRNAs extensively involved in crucial biological processes such as cell growth and development but they also participate in the occurrence and development of various diseases such as osteosarcoma, Alzheimer’s disease, and myocardial infarction (Han et al., 2020; Huang et al., 2020; Yang et al., 2020). In recent studies, several lncRNAs, such as linc-MD1 (Cesana et al., 2011; von Roretz et al., 2011), Yam-1 (Lu et al., 2013), Linc-RAM (Yu et al., 2017), lncMyoD (Gong et al., 2015), and lnc-mg (Zhu et al., 2017), were shown to affect the proliferation and differentiation of skeletal muscle cells through various mechanisms.

We previously discovered dozens of uncharacterized lncRNAs from mouse C2C12 myoblast differentiation versus myoblast proliferation using gene chip analysis. A co-expression network revealed that one lncRNA, Gm13398, exhibited relatively high expression and was co-expressed with Myog during myoblast differentiation (Chen et al., 2018). In the present study, Gm13398 was named the lncRNA of growth and differentiation to histogenesis (lnc-GD2H) in myogenic cells and was shown to significantly contribute to C2C12 myoblast proliferation and differentiation. Consistently, small interfering RNA (siRNA) of lnc-GD2H impaired muscle regeneration after cardiotoxin (CTX)-induced injury in mice. Further research showed that lnc-GD2H could promote proliferation by forming a feedback loop with c-Myc and enhance differentiation through interacting with NACA to upregulate Myog. These results have significantly expanded our understanding of the mechanisms by which lncRNAs govern myoblast proliferation and differentiation and muscle regeneration and provide a preliminary experimental basis for promoting muscle regeneration as well as novel ideas and therapeutic targets for the regulation of lncRNA involvement in muscle regeneration.

## Materials and Methods

### Cell Lines

Mouse C2C12 myoblasts and 293T cells (Shanghai Cell Bank of the Chinese Academy of Sciences, China) were cultured in Dulbecco’s modified Eagle medium (DMEM) (cat. no. 12100046; Gibco, United States) supplemented with 10% fetal bovine serum (cat. no. 10270-106; Gibco) at 37°C in 5% CO_2_. For the myogenic differentiation experiment, C2C12 myoblasts were seeded in six-well plates, and when they reached 80–90% confluence, they were placed in DMEM containing 2% horse serum (cat. no. 16050-114; Gibco).

### RNA Extraction and Quantitative Polymerase Chain Reaction (qPCR) Analysis

Total RNA was extracted from cultured cells or tissues using TRIzol reagent (Takara, Japan) according to the manufacturer’s protocol. Complementary DNAs were synthesized from 500 ng of total RNA using a Prime Script RT reagent kit (cat. no. RR036A; Takara). The quantitative polymerase chain reaction (qPCR) assays were performed in the StepOnePlus^TM^ Real-Time PCR System (Applied Biosystems, United States) using SYBR-Green Mix (cat. no. RR820A; Takara), with the expression levels normalized over an 18s period. Relative RNA expression was calculated using the 2^–ΔΔCt^ method. The primer sequences are listed in Supplementary Table 1.

### Fluorescence *in situ* Hybridization (FISH)

Oligonucleotides (Bersin Bio, China) targeting lnc-GD2H were modified with fluorescein isothiocyanate. Briefly, for lnc-GD2H fluorescence *in situ* hybridization (FISH), cells were fixed, permeabilized, and hybridized with lnc-GD2H probe in buffer overnight at 42°C. Then, nuclei were stained with DAPI. Images were captured using a laser scanning confocal microscope (LSM 880; Carl Zeiss, Germany) at 37°C.

### Western Blotting Analysis

Total protein was extracted using RIPA lysis buffer containing phenylmethylsulfonyl fluoride, then subjected to sodium dodecyl sulfate-polyacrylamide gel electrophoresis (SDS-PAGE) and transferred to polyvinylidene difluoride membranes (Merck Millipore, United States). The membranes were incubated with primary antibodies against Tubulin (1:5,000; cat. no. AC021; ABclonal, United States), CDK2 (1:1,000; cat. no. A18000; ABclonal), CDK4 (1:1,000; cat. no. A11136; ABclonal), CDK6 (1:1,000; cat. no. A1545; ABclonal), c-Myc (1:1,000; 9042; Cell Signaling Technology, United States), myosin heavy chain (MyHC) (1:1,000; cat. no. MAB4470; R&D Systems, United States), Myog (1:1,000; cat. no. MAB3876; Millipore), Mef2a (1:20,000; cat. no. ab109420; Abcam, United Kingdom), Mef2c (1:1,000; cat. no. 5030; Cell Signaling Technology), and NACA (1:1,000; cat. no. A10122; ABclonal). The membranes were then incubated with horseradish peroxide-conjugated goat anti-mouse (1:10,000; cat. no. AS003; ABclonal) or anti-rabbit (1:10,000; cat. no. AS014; ABclonal) secondary antibody. Band intensities were determined using a chemiluminescent imaging system (Tanon-V8 Pro; Tanon Science and Technology, China).

### Overexpression of Lnc-GD2H and c-Myc Using Stable Cell Line Generation With Lentivirus Infection

C2C12 myoblasts were infected with negative control lentiviruses (lv-OE-NC) or those containing lnc-GD2H (lv-OE-lnc-GD2H) or c-Myc (lv-OE-c-Myc) at a multiplicity of infection of 100 following the manufacturer’s instructions. The lv-OE-NC, lv-OE-lnc-GD2H, and lv-OE-c-Myc lentiviruses were synthesized by Hanbio (China). Then, stably transfected cells were selected and maintained using puromycin (cat. no. MB2500; Meilun Bio, China).

### siRNAs

siRNAs against lnc-GD2H, c-Myc, and NACA were synthesized by Gene Pharma (China). For siRNA transfection, cells were transfected with the indicated siRNAs using Lipofectamine^TM^ RNAi MAX (13778150; Invitrogen, United States), following the manufacturer’s instructions.

### Determination of Cell Proliferation

#### CCK-8 Assay

Cell proliferation was assessed using the Cell Counting Kit-8 (CCK-8) assay (C0038; Beyotime Institute of Biotechnology, China). Stably transfected lv-OE-NC, lv-OE-lnc-GD2H, and lv-OE-c-Myc overexpression myoblasts were cultured in 96-well plates. siRNA-transfected NC (siNC), lnc-GD2H, and c-Myc myoblasts were also cultured in 96-well plates. After 24, 48, or 72 h of siRNA and overexpression, CCK-8 reagent was added to each well for 1 h at 37°C. Then, the absorbance was measured at 450 nm using an automatic microplate reader (TAKE3; Biotech, United States).

#### EdU Proliferation Assay

For 5-ethynyl-2′-deoxyuridine (EdU) staining, stably transfected lv-OE-NC, lv-OE-lnc-GD2H, and lv-OE-c-Myc overexpression myoblasts were cultured in 96-well plates. siRNA-transfected NC, lnc-GD2H, and c-Myc myoblasts were also cultured in 96-well plates. After 72 h of siRNA and overexpression, EdU reagent (cat. no. KGA337; KeyGen Biotech, China) was added at a final concentration of 10 μM and the plates incubated for 2 h at 37°C. Then, cells were harvested for EdU staining according to the manufacturer’s instructions.

### Immunofluorescence Staining and Fusion Index

First, lnc-GD2H-overexpressing and control stable cell lines were cultured in 96-well plates and allowed to differentiate for 72 h. The harvested cells were fixed, blocked with goat serum (cat. no. C0265; Beyotime Institute of Biotechnology), incubated with primary anti-MyHC antibody (1:80; cat. no. MAB4470; R&D Systems), and then incubated with Alexa Fluor 594-conjugated AffiniPure goat anti-mouse IgG secondary antibody (1:100; cat. no. AS077; ABclonal). DAPI (cat. no. KGA215; KeyGen Biotech) was used to stain the cell nuclei. Images were captured using a Leica DMIL LED fluorescence microscope (Leica Microsystems GmbH, Germany). Fusion indices were calculated as the percentage of nuclei with two or more nuclei in the fused myotubes in MyHC-positive cells out of the total nuclei (Sui et al., 2019).

### Luciferase Reporter Assay

The c-Myc plasmid together with the wild-type lnc-GD2H promoter P1 (at the approximate site −1 to −2,000), the truncated lnc-GD2H promoter P2 (at the approximate site −1 to −1,400), three predicted simultaneously mutated c-Myc binding sites in the lnc-GD2H promoter (at the approximate site −1,400 to −2,000), or the NACA and lnc-GD2H plasmid together with the Myog promoter were transfected into 293T cells using FItran transfection reagent (cat.no. FG005, FitGene Biotechnology, China) in 96-well plates. The potential binding sites of NACA in the Myog promoter region were mutated from GGAGAGAGTAG to CCTCTCTCATC at the site −331 to −341 (Myog promoter-Mut1) and from GCACAGAAGAG to CGTGTCTTCTC at −1,356 to −1,366 (Myog promoter-Mut2). Firefly activity observed with the Dual-Luciferase^®^ Reporter Assay System (Promega, United States) was normalized to *Renilla* luciferase activity. The data are presented as the mean ± standard deviation (SD) of three independent experiments.

### DNA Pulldown

Linearizing DNA was biotin-labeled and incubated with proteins extracted from C2C12 overnight at room temperature. Next, streptavidin beads (cat. no. 21344; Thermo Fisher Scientific) were added to each reaction mixture, which were then incubated for 2 h at 4°C. Finally, the beads were washed in buffer (cat.no. FP1822; FitGene Biotechnology), and the bound proteins were used for western blotting.

### Chromatin Immunoprecipitation (ChIP) Assay

The ChIP assay was performed using the ChIP kit (cat. no. FI88806-1, FitGene Biotechnology) according to the manufacturer’s instructions. Briefly, DNA that had undergone ChIP was eluted, reverse X-linked, and purified. Each ChIP reaction was performed using 1 μg of NACA antibody (A10122; ABclonal), and IgG was applied as the isotype control. Fold enrichment was quantified using qPCR.

### RNA Pulldown

Briefly, vectors carrying lnc-GD2H and antisense lnc-GD2H were linearized with the corresponding restriction enzymes to prepare template DNAs for *in vitro* transcription. Biotinylated RNAs were mixed with proteins extracted from C2C12 cells, followed by targeting of the RNAs with streptavidin beads (cat. no. 21344; Thermo Fisher Scientific). The co-precipitated proteins were used for mass spectrometry.

### Mass Spectrometry (MS)

After SDS-PAGE following RNA pulldown, the differentially expressed and obviously present band in the Coomassie brilliant blue staining lane was excised and used for MS analysis. The UniProt database was used for identifying proteins. Proteins were identified and ranked based on percentage cover (i.e., the number of detected amino acids based on MS/the total number of amino acids in the protein) and the number of unique peptides. The MS proteomics data have been deposited to the ProteomeXchange Consortium via the PRIDE partner repository with the dataset identifier PXD025292.

### RNA Immunoprecipitation (RIP)

RNA immunoprecipitation assay was conducted using RIP Kit (cat. no. FI88806-2, FitGene Biotechnology) according to the manufacturer’s instructions. Briefly, C2C12 cells were lysed with RIP lysis buffer, and incubated with the antibody against NACA (A10122; ABclonal) for RIP. Then Protein A/G beads were added to the lysates to pull down antibody-protein-RNA complex. The co-precipitated RNAs were analyzed using qPCR. Total RNAs (input control) and the negative control (IgG) were assayed at the same time to confirm that the examined signals were from RNAs binding to NACA.

### Wound Healing Assay

Stably transfected lv-OE-NC or lv-OE-lnc-GD2H C2C12 overexpression myoblasts and siNC or silnc-GD2H myoblasts were cultured in six-well plates and then serum-starved for 24 h. Next, artificial wounds were scratched on the monolayers across the well using 10 μL pipette tips, and the scratched areas were photographed every 6 h until 24 h after wounding. The wound size was determined by calculating the ratio between the surface area of the wound after scratch of 6 h, 12 h, 24 h and the area of the initial wound (0 h).

### Muscle Injury and Regeneration

Six-week male C57BL/6 mice were purchased from the Animal Laboratory of Sun Yat-sen University (Guangzhou, China). The study was approved by the Ethics Committee of Guangdong Second Provincial General Hospital (no: 2017-YJS-033). For CTX injection, muscle injury was induced by injecting 100 μL of 10 μM CTX (dissolved in PBS) into the right tibialis anterior muscles of the mice on day 0. Then, 2.5 nmol silnc-GD2H/siNC oligonucleotides were prepared by pre-incubating with Lipofectamine 2000 for 15 min and injected on days −1, 1, and 4. The muscles of six mice were harvested on days 3 and 6, and RNA and proteins were then extracted for qPCR, western blotting, and hematoxylin and eosin (H&E) staining. Mice were sacrificed *via* the inhalation of isoflurane, and all efforts were made to minimize suffering.

### H&E Staining and Muscle Fiber Cross-Sectional Area (CSA) Determination

To assess tissue morphology, freshly isolated tibialis anterior muscles were fixed with 4% paraformaldehyde and stained with H&E. Next, the sections were analyzed under a microscope (DMIL; Leica). Six microscope images per field were captured. Muscle fiber cross-sectional area (CSA) in the images was measured using ImageJ software (v1.44P; National Institutes of Health, United States).

### Online Tools Used for Prediction

The transcription factor prediction database JASPAR^1^ was used to predict the c-Myc binding sites in the lnc-GD2H promoter sequence. The universal protein knowledge base UniProt^2^ was used to retrieve the proteins identified based on MS. The online RNA-protein interaction prediction tool RPISeq^3^ was used to predict the possible interactions between lnc-GD2H and NACA; random forest (RF) classifier and support vector machine (SVM) classifier values greater than 0.5 were considered “positive.”

### Statistical Analysis

All experimental values are presented as the mean ± SD in graphs plotted using GraphPad Prism 5.0 (GraphPad Software, United States). Comparisons between two groups were performed using the *t*-test. A *P*-value < 0.05 was considered to indicate statistical significance.

## Results

### Expression Patterns and RNA FISH Localization of Lnc-GD2H in Myoblast Proliferation and Differentiation

**FIGURE 1:**
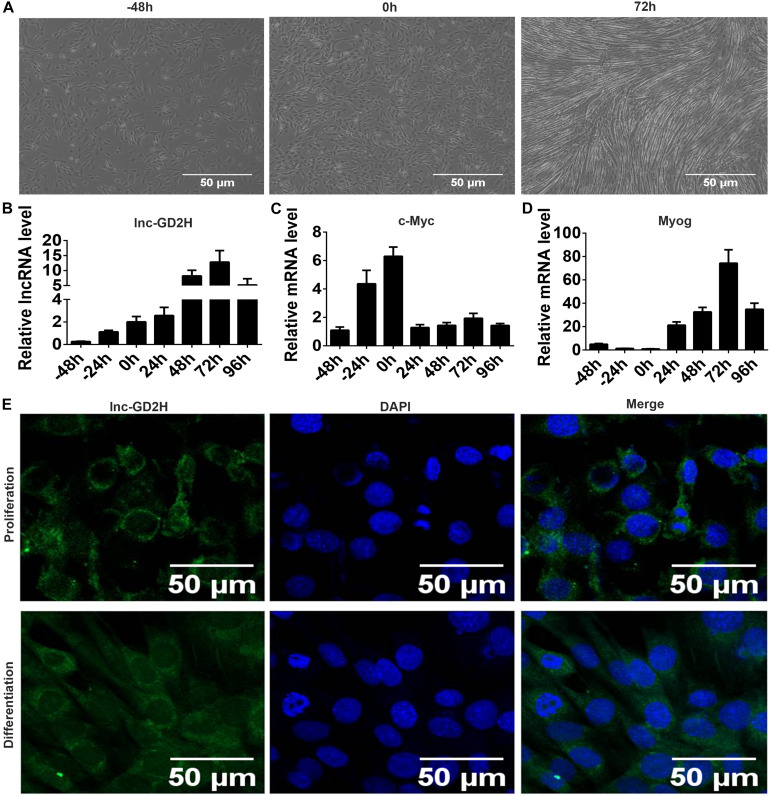
Expression patterns and FISH localization of lnc-GD2H during myoblast proliferation and differentiation. **(A)** Representative photos of C2C12 cells at –48, 0, and 72 h during proliferation and differentiation. **(B)** lnc-GD2H expression level during myoblast proliferation and differentiation. **(C)** c-Myc expression level during myoblast proliferation and differentiation. **(D)** Myog expression level during myoblast proliferation and differentiation. **(E)** RNA FISH for detecting lnc-GD2H in C2C12 cells. Green: lnc-GD2H, blue: DAPI staining, *n* = 3.

To explore the role of lnc-GD2H in myogenesis, we first observed the cell morphology under a microscope (Figure 1A) and detected the expression patterns of lnc-GD2H, c-Myc, and Myog during C2C12 myoblast proliferation and differentiation by qPCR. Expression level of lnc-GD2H gradually increased during proliferation and differentiation (Figure 1B). c-Myc increased during proliferation, but it decreased after the beginning of differentiation (Figure 1C). Myog was significantly upregulated during differentiation (Figure 1D). The results indicated that lnc-GD2H may be a myogenic factor in proliferation and differentiation. RNA FISH showed that lnc-GD2H was distributed in the cytoplasm and nucleus during myoblast proliferation and differentiation (Figure 1E).

### Lnc-GD2H Promoted Cell Proliferation and Enhanced c-Myc Expression During C2C12 Myoblast Proliferation

**FIGURE 2:**
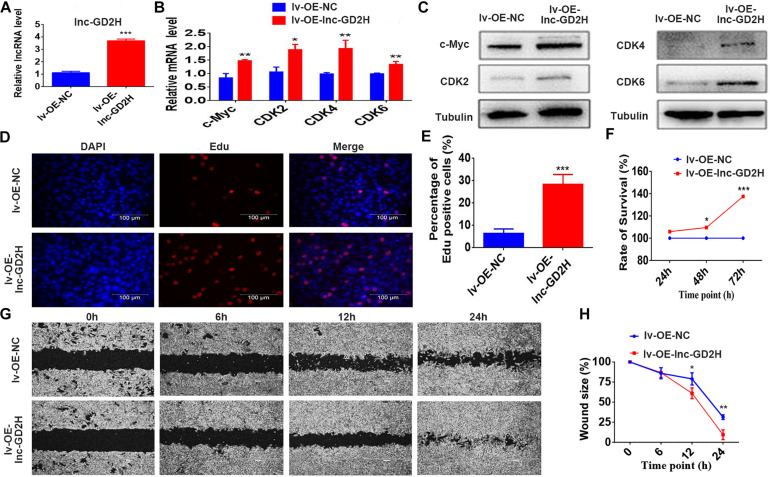
Overexpression of lnc-GD2H promoted proliferation and enhanced c-Myc expression. **(A)** lnc-GD2H relative expression level in lnc-GD2H-overexpressing C2C12 myoblasts infected with lentivirus. **(B,C)** Expression levels of the proliferation marker genes c-Myc, CDK2, CDK4, and CDK6 in lnc-GD2H-overexpressing myoblasts were quantified using qPCR and western blotting. **(D)** Analysis of cell proliferation using an EdU assay in lnc-GD2H-overexpressing myoblasts. **(E)** Percentage of EdU-positive cells in lnc-GD2H-overexpressing myoblasts. **(F)** Cell survival was analyzed using the CCK-8 assay in lnc-GD2H-overexpressing myoblasts. **(G)** Wound healing assay of lnc-GD2H-overexpressing myoblasts. **(H)** Percentage wound size after scraching was shown in lnc-GD2H-overexpressing myoblasts. *n* = 3, *P < 0.05, **P < 0.01, and ***P < 0.001.

Mouse C2C12 myoblasts were selected to investigate the potential role of lnc-GD2H in myogenesis. Stably transfected cells were selected to examine the overexpression effect of lnc-GD2H. The qPCR results showed lnc-GD2H expression was significantly activated in lnc-GD2H stably transfected lentivirus-infected myoblast cells (lv-OE-lnc-GD2H) compared with control myoblast cells (lv-OE-NC) during myoblast proliferation (Figure 2A). Different experimental approaches, including qPCR, western blotting, EdU, CCK-8 assays, and wound healing assay were used to determine the potential function of lnc-GD2H in myoblast proliferation. The results indicated that the mRNA expression and protein levels of various proliferation markers including c-Myc, CDK2, CDK4, and CDK6 (Figures 2B,C) were high in lv-OE-lnc-GD2H myoblast cells compared with lv-OE-NC myoblast cells. Based on EdU analysis, the percentage of EdU-positive cells was higher in lv-OE-lnc-GD2H cells than in lv-OE-NC cells (Figures 2D,E). Furthermore, the CCK-8 assay was performed to investigate the contribution of lnc-GD2H to cell growth and division. The analysis showed that lnc-GD2H-transfected cells had a high rate of cell survival compared with the control cells (Figure 2F). The wound size inflicted in lnc-GD2H-transfected cells were significantly smaller than in lv-OE-NC cells after 12 and 24 h of forming scratch wounds (Figures 2G,H), indicating that lnc-GD2H overexpression promoted the proliferation and migratory potential of C2C12 myoblasts.

**FIGURE 3:**
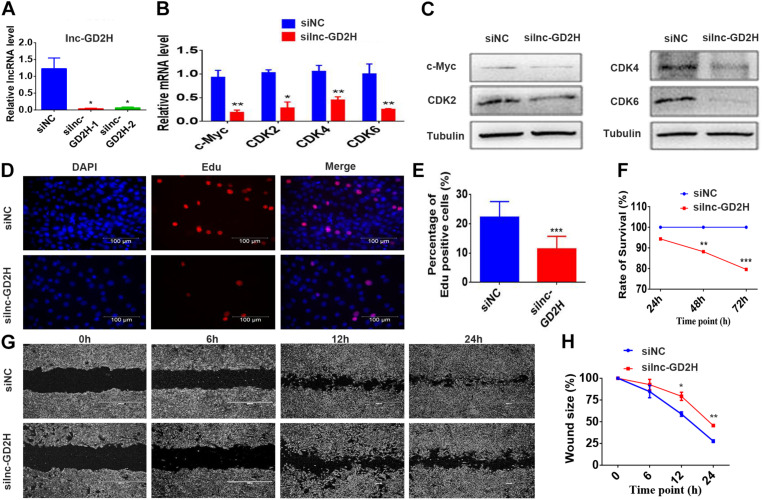
Knockdown of lnc-GD2H inhibited proliferation and decreased c-Myc expression. **(A)** Relative lnc-GD2H expression level in C2C12 myoblasts expressing siNC or silnc-GD2H as confirmed using qPCR. **(B,C)** Expression levels of c-Myc, CDK2, CDK4, and CDK6 in lnc-GD2H-knockdown myoblasts as examined using qPCR and western blotting. **(D)** Cell proliferation was investigated using the EdU assay in lnc-GD2H-knockdown myoblasts. **(E)** Percentage of EdU-positive cells in lnc-GD2H-knockdown myoblasts. **(F)** Cell survival was determined using the CCK-8 assay in lnc-GD2H-knockdown myoblasts. **(G)** Wound healing assay of lnc-GD2H-knockdown myoblasts. **(H)** Percentage wound size after scraching was shown in lnc-GD2H-knockdown myoblasts. *n* = 3, *P < 0.05, **P < 0.01, and ***P < 0.001.

Conversely, knockdown of lnc-GD2H with two independent siRNAs (Figure 3A) inhibited the proliferation of C2C12 myoblasts with reduced c-Myc, CDK2, CDK4, and CDK6 mRNA (Figure 3B) and protein (Figure 3C) levels, a decreased percentage of EdU-positive cells (Figures 3D,E), and a lower rate of cell survival (Figure 3F). The wound size in silnc-GD2H cells were significantly larger than in siNC cells of 12 and 24 h after scratching (Figures 3G,H), indicating that lnc-GD2H knockdown restrained myoblast proliferation and migration. Based on these results, lnc-GD2H was proven essential for C2C12 myoblast proliferation and may promote c-Myc expression.

### c-Myc Regulated the Transcription of Lnc-GD2H During Myoblast Proliferation

**FIGURE 4:**
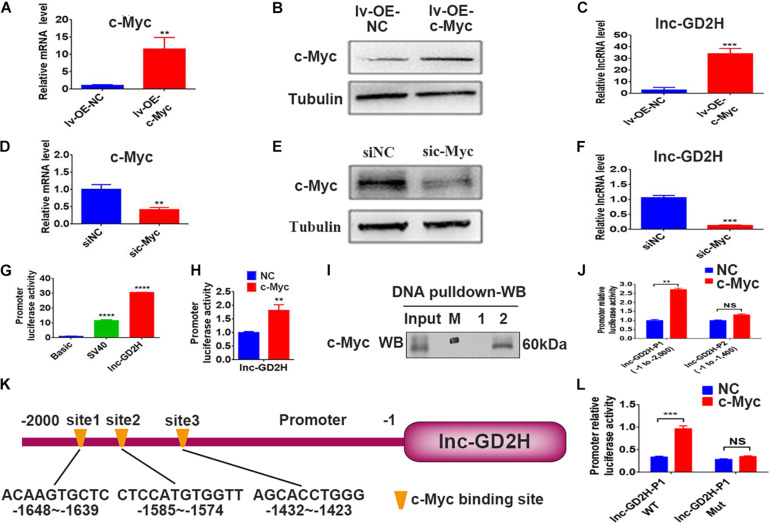
c-Myc regulated the transcription of lnc-GD2H during myoblast proliferation. **(A)** c-Myc mRNA and **(B)** protein levels in c-Myc-overexpressing C2C12 cells. **(C)** Level of lnc-GD2H in c-Myc-overexpressing cells. **(D,E)** qPCR and western blotting confirmed the knockdown of c-Myc. **(F)** Level of lnc-GD2H in c-Myc-knockdown cells. **(G)** Luciferase activity of the lnc-GD2H promoter. **(H)** Luciferase activity of the lnc-GD2H promoter after transfection with c-Myc plasmid and the lnc-GD2H promoter. **(I)** A DNA pulldown assay was performed to confirm the association between the lnc-GD2H promoter and c-Myc. M: marker, 1: biotinylated control probe, 2: biotinylated lnc-GD2H-probe. **(J)** Luciferase activity of lnc-GD2H after wild-type promoter P1 (at approximate site –1 to –2,000) or promoter P2 (at approximate site –1 to –1,400) was transfected with c-Myc plasmid. **(K)** The JASPAR database was used to predict the promoter sequence lnc-GD2H binding site for c-Myc. **(L)** Luciferase activity of the wild-type lnc-GD2H promoter and three predicted simultaneously mutated c-Myc binding sites in the lnc-GD2H promoter, for determining the binding domain between the lnc-GD2H promoter and c-Myc. *n* = 3, **P < 0.01, ***P < 0.001, ****P < 0.0001, NS: non-significant.

Recently, c-Myc was reported to bind to and control dozens of lncRNAs to regulate skeletal muscle development (Luo et al., 2019). In the present study, mRNA (Figure 4A) and protein expression (Figure 4B) analyses confirmed that the overexpression of c-Myc (lv-OE-c-Myc) leads to an increased percentage of EdU-positive cells (Supplementary Figures 1A,B), a significant acceleration in cell survival rate (Supplementary Figure 1C), and a higher lnc-GD2H level (Figure 4C). Conversely, c-Myc knockdown (Figures 4D,E) lowered the percentage of EdU-positive cells (Supplementary Figures 1D,E), inhibited the cell survival rate (Supplementary Figure 1F), and decreased the lnc-GD2H level (Figure 4F).

Luciferase reporter experiments showed that c-Myc enhanced the promoter activity of lnc-GD2H (Figures 4G,H). Therefore, we hypothesized that c-Myc promotes myoblast proliferation by regulating lnc-GD2H transcription. Next, DNA pulldown and western blotting were performed to identify the c-Myc protein that had bound to the lnc-GD2H promoter during C2C12 myoblast proliferation (Figure 4I). c-Myc enhanced the luciferase activity of the lnc-GD2H wild-type promoter P1 (at the approximate site −1 to −2,000) but could not facilitate the activity of the lnc-GD2H promoter P2 (at the approximate site −1 to −1,400), indicating that the c-Myc binding site existed at the approximate location of −1,400 to −2,000 in the lnc-GD2H promoter (Figure 4J). The JASPAR database was used to analyze the lnc-GD2H promoter sequence, and three binding sites were predicted for the c-Myc transcription factor (Figure 4K). The three predicted c-Myc binding sites on the lnc-GD2H promoter were simultaneously mutated, and c-Myc had no obvious promotional effect on the activity of the lnc-GD2H mutated promoter (Figure 4L), indicating that c-Myc can bind to these sites on the lnc-GD2H promoter.

### Lnc-GD2H Promoted Cell Differentiation During Myoblast Differentiation

**FIGURE 5:**
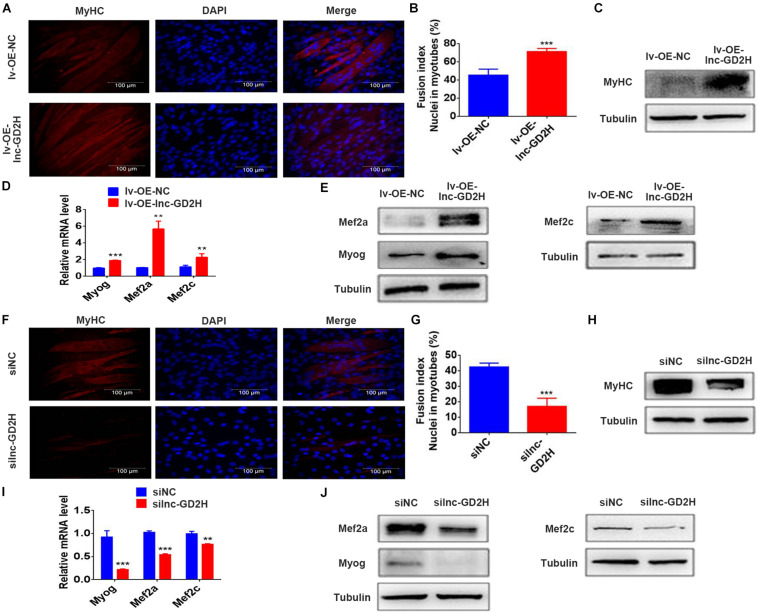
Lnc-GD2H promoted cell differentiation during myoblast differentiation. The differentiation of lnc-GD2H-overexpressing C2C12 cells in differentiation medium was detected by staining for MyHC **(A,B)** the fusion index was calculated. **(C)** MyHC protein levels were detected using western blotting. The mRNA levels **(D)** and protein levels **(E)** of Myog, Mef2a, and Mef2c were detected using qPCR and western blotting in lnc-GD2H-overexpressing C2C12 cells. Knockdown of lnc-GD2H decreased myogenic differentiation as evidenced by less MyHC staining **(F)**, a reduced fusion index **(G)**, lower MyHC protein levels **(H)**, and decreased mRNA **(I)** and protein levels **(J)** of Myog, Mef2a, and Mef2c. *n* = 3, **P < 0.01, and ***P < 0.001.

Based on the lnc-GD2H expression profile during C2C12 myoblast differentiation, lnc-GD2H also potentially played a regulatory role in modulating myoblast differentiation. To investigate whether lnc-GD2H affects myoblast differentiation, immunofluorescence staining, qPCR and western blotting were used to analyze myoblast differentiation after lnc-GD2H overexpression or knockdown. The results showed that overexpression of lnc-GD2H led to a significant acceleration in C2C12 differentiation based on enhanced immunostaining and higher protein levels of MyHC (Figures 5A,C), which is a terminal differentiation marker, an accelerated fusion index (Figure 5B), and increased expression of the myogenic marker genes Myog, Mef2a, and Mef2c (Figures 5D,E). Conversely, knockdown of lnc-GD2H repressed C2C12 myoblast differentiation with decreased MyHC immunostaining and protein levels (Figures 5F,H), a reduced fusion index (Figure 5G), and weakened expression of Myog, Mef2a, and Mef2c (Figures 5I,J). Taken together, the results showed that lnc-GD2H promoted myogenic differentiation.

### Lnc-GD2H Interacted With NACA

**FIGURE 6:**
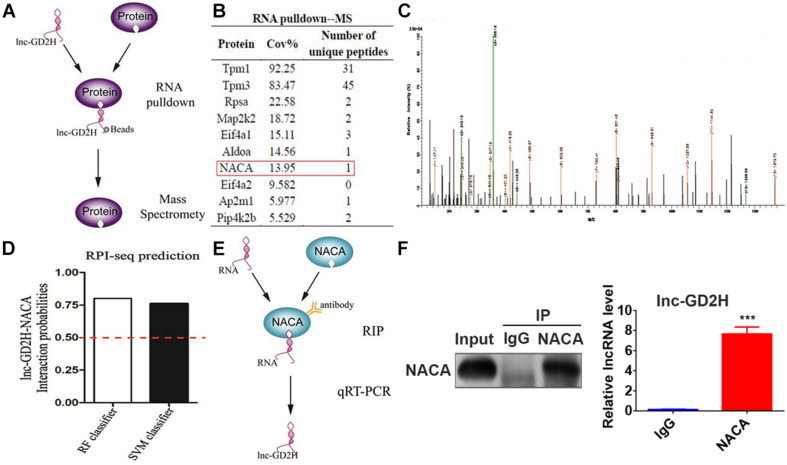
Lnc-GD2H interacted with NACA. **(A)** A schematic representation of the RNA pulldown and MS assays. **(B)** The partial list of proteins detected using MS. **(C)** The NACA protein was successfully identified using MS. **(D)** A probable interaction between lnc-GD2H and NACA was predicted using the online tool RPISeq based on RF and SVM classifiers (probabilities >0.5 were considered “positive”). **(E)** A schematic representation of the RIP assay. **(F)** RIP assay performed in C2C12 cells using NACA antibodies. The retrieved lnc-GD2H transcripts were assessed by qPCR. The RIP assay indicated an interaction between NACA and lnc-GD2H based on qPCR. ***P < 0.001.

Next, the molecular mechanisms underlying the promotional role of lnc-GD2H in myoblast differentiation were investigated. RNA FISH with differentiating C2C12 cells indicated that lnc-GD2H was distributed in the cytoplasm and nucleus (Figure 1D). Many nuclear lncRNAs interact with other proteins (Huarte et al., 2010). To dissect the molecular mechanism, we sought to identify the interacting protein partners of lnc-GD2H. Therefore, a biotin RNA pulldown assay was performed to identify the proteins associated with lnc-GD2H (Figure 6A). Biotinylated lnc-GD2H and antisense lnc-GD2H were transcribed *in vitro*, incubated with proteins extracted from C2C12, captured by streptavidin beads, and then subjected to SDS-PAGE analysis. The same proteins from SDS-PAGE were then used for MS (Q Exactive quadrupole Orbitrap, Thermo Fisher Scientific, United States) analysis, and the specific proteins binding to lnc-GD2H were detected. Proteins that potentially interact with lnc-GD2H detected using MS are listed in Figure 6B. NACA was successfully identified using MS (Figure 6C), which was of particular interest because it acts as a key mediator of cell developmental processes such as differentiation (Moreau et al., 1998; Lopez et al., 2005; Jafarov et al., 2012).

In addition, we also analyzed the possible interaction between lnc-GD2H and NACA using the online database RPISeq; the results showed that lnc-GD2H would very likely bind to NACA based on RF classifier and SVM classifier values greater than 0.5 (Figure 6D). To further validate the interaction between lnc-GD2H and NACA, a RIP assay with C2C12 cell extracts was performed (Figures 6E,F). Next, qPCR analysis of antibody-enriched RNA showed that the NACA antibody pulled down significantly more lnc-GD2H than did the IgG control (Figure 6F). These findings indicate that lnc-GD2H interacts with NACA.

### Lnc-GD2H Decreased the Enrichment of NACA in the Myog Promoter

**FIGURE 7:**
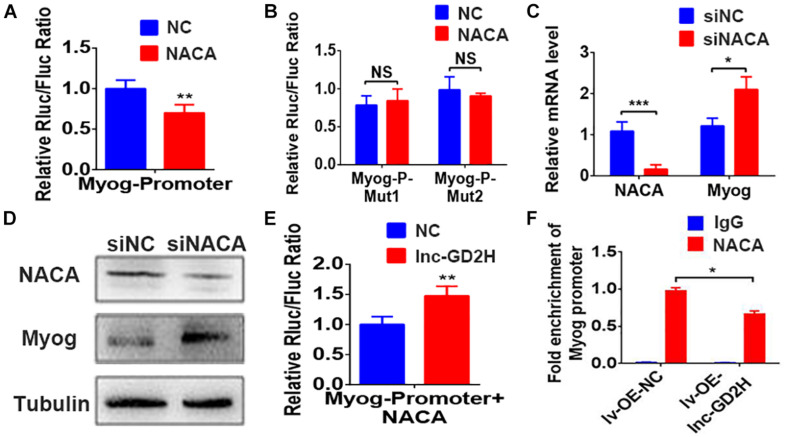
Lnc-GD2H decreased the enrichment of NACA in the Myog promoter. NACA overexpression inhibited the luciferase activity of the wild-type Myog promoter construct **(A)** but not that of the mutant Myog promoter reporter construct **(B)**. **(C)** Myog mRNA and **(D)** protein levels after NACA knockdown. **(E)** Luciferase activity upon lnc-GD2H overexpression after NACA and the lnc-GD2H plasmid were transfected together with the Myog promoter. **(F)** Enrichment of NACA at the promoter region of Myog after lnc-GD2H overexpression. *n* = 3, *P < 0.05, **P < 0.01, and ***P < 0.001. NS: non-significant.

Next, we predicted there were two NACA DNA binding sites located in the promoter region of the Myog gene based on a previous report (Yotov and St-Arnaud, 1996). The luciferase reporter assay indicated that NACA significantly decreased the luciferase activity of the wild-type Myog promoter construct (Figure 7A) but not that of the mutant Myog promoter reporter construct (Figure 7B). These results indicate that Myog promoter-Mut1 and Myog promoter-Mut2 mutation sites may be the NACA binding sites. Furthermore, knockdown of NACA enhanced Myog mRNA (Figure 7C) and protein levels (Figure 7D).

There was no significant difference in the expression of NACA during myoblast proliferation and differentiation (Supplementary Figure 2). Overexpression of lnc-GD2H reversed the decreased Myog promoter luciferase activity caused by NACA overexpression (Figure 7E). Furthermore, ChIP-PCR revealed that the enrichment of NACA at the Myog promoter region was significantly impaired by lnc-GD2H overexpression (Figure 7F). These findings showed that lnc-GD2H can bind to the transcription factor NACA to relieve the inhibitory effect of NACA on Myog and improve Myog transcription to promote myoblast differentiation.

### Lnc-GD2H Promoted the Repair of Muscle Injury

**FIGURE 8:**
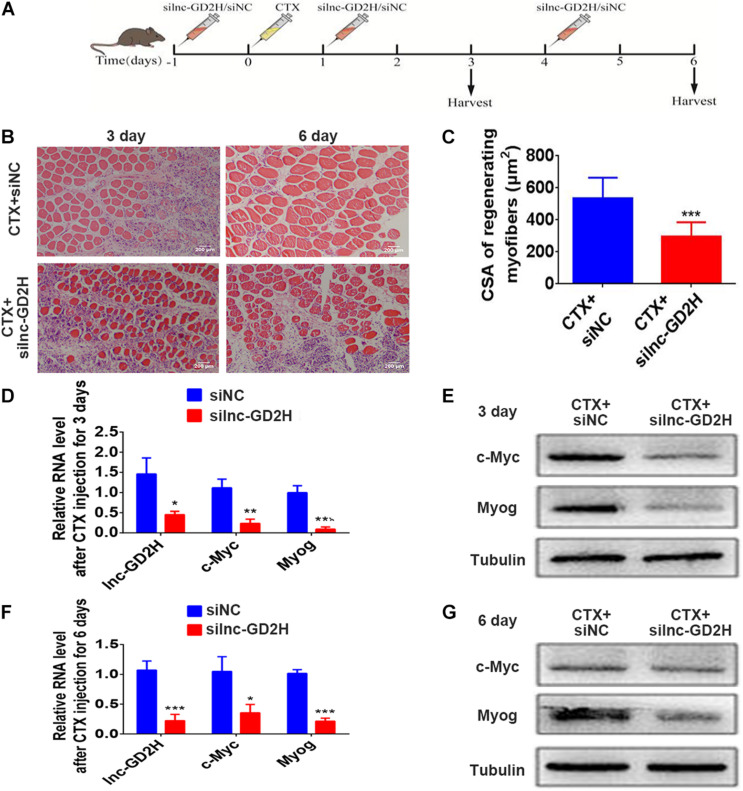
Lnc-GD2H promoted the repair of muscle injury. **(A)** Protocol for siNC or silnc-GD2H injection into CTX-injured muscles. **(B)** Representative H&E-stained images of the tibialis anterior muscle on days 3 and 6 after CTX injury. **(C)** The average fiber CSA on day 6 after CTX injury. **(D)** Relative expression level of lnc-GD2H on day 3 after CTX injection based on qPCR; knockdown of lnc-GD2H reduced the mRNA **(D)** and protein **(E)** levels of c-Myc and Myog on day 3 after CTX injection. **(F)** Relative lnc-GD2H expression level on day 6 after CTX injection; knockdown of lnc-GD2H reduced the mRNA **(F)** and protein **(G)** levels of c-Myc and Myog on day 6 after CTX injection. Animal study, *n* = 6; cell experiment, *n* = 3. *P < 0.05, **P < 0.01, and ***P < 0.001.

The above findings emphasize the role of lnc-GD2H in myoblast proliferation and differentiation, indicating that lnc-GD2H may have an effect on muscle regeneration. Next, lnc-GD2H was depleted in the hind leg muscles of mice during an *in vivo* assay of injury-induced regeneration. First, silnc-GD2H and siNC were injected three times, on the day before CTX injection (−1), and on days 1 and 4 post-CTX injection. The muscles were then harvested at the assigned times for analyses as illustrated (Figure 8A). CTX induced muscle damage in both silnc-GD2H- and siNC-injected muscles on days 3 and 6 after injury (Figure 8B). Regenerating myofibers, characterized by centralized nuclei, were significantly smaller in the silnc-GD2H-injected muscles than in siNC-injected muscles with H&E staining on days 3 and 6 after CTX injection (Figure 8B), and their average CSA on day 6 after CTX injury was smaller as well (Figure 8C). We found that the silnc-GD2H injection resulted in significant loss of lnc-GD2H expression based on qPCR on day 3 after injury (Figure 8D). Consistently, the mRNA (Figure 8D) and protein (Figure 8E) expression levels of c-Myc and Myog were significantly reduced on day 3 after injury based on qPCR and western blotting. Similar results were obtained on day 6 after injection (Figures 8F,G). Altogether, the results showed that the loss of lnc-GD2H led to a significant delay in injury-induced muscle regeneration, indicating that lnc-GD2H may promote the repair of muscle injury and muscle regeneration.

## Discussion

**FIGURE 9:**
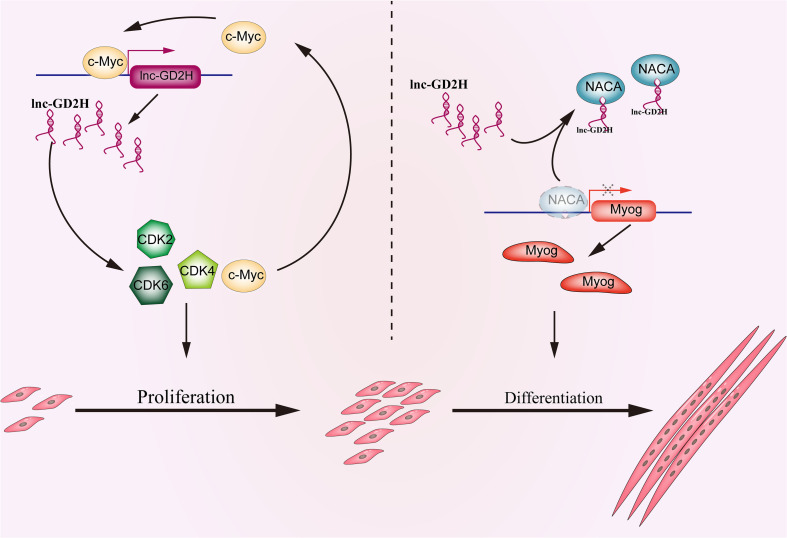
Schematic depicting the functional roles of lnc-GD2H during myoblast proliferation and differentiation. In proliferating myoblasts, lnc-GD2H facilitates the expression of proliferating marker genes and forms a feedback loop with c-Myc. In differentiating myoblasts, lnc-GD2H binds to NACA to relieve the inhibitory effect of NACA on Myog to improve the Myog transcription and promote myoblast differentiation.

Skeletal muscle development is tightly regulated by a complex network. Increasing evidence indicates that lncRNAs may play a crucial role in regulating myogenesis (Ballarino et al., 2018; Jin et al., 2018; Militello et al., 2018; Zhang et al., 2018c; Li et al., 2019, 2020; Sui et al., 2019). In the present study, the biological function of a novel lncRNA, lnc-GD2H, in regulating myoblast proliferation and differentiation and muscle regeneration was determined (Figure 9). Collectively, our findings indicate that lnc-GD2H facilitates the expression of proliferating marker genes and forms a feedback loop with c-Myc during myoblast proliferation. We found that lnc-GD2H bound to NACA to relieve the inhibitory effect of NACA on Myog, subsequently promoting Myog expression during myoblast differentiation.

NACA was initially reported to bind to newly synthesized polypeptides emerging from ribosomes in yeast and higher eukaryotes (Wiedmann et al., 1994; Lauring et al., 1995). Further studies indicated that NACA functions as a transcriptional co-activator in osteoblasts by interacting with phosphorylated c-Jun (Moreau et al., 1998) or GAL4/VP-16 and TATA box-binding proteins (Yotov et al., 1998). In recent studies, NACA played a significant role in regulating the differentiation of erythroid cells, osteoblasts, and myoblasts (Lopez et al., 2005; Jafarov et al., 2012; Addison et al., 2019). In the present study, lnc-GD2H bound to the NACA transcription factor to relieve the inhibitory effect of NACA on Myog. The function of NACA in this study was consistent with the role of NACA during myoblast differentiation in a previous study, where NACA overexpression in myoblasts inhibited Myog expression and differentiation, and overexpression of an N-terminal-truncated NACA mutant allowed myoblasts to express Myog and differentiate (Jafarov et al., 2012).

Myogenin is an important positive regulator in muscle development. Reportedly, miRNAs and lncRNAs can regulate Myog to affect myoblast differentiation (Cesana et al., 2011; Zhou et al., 2015; Zhu et al., 2017); however, the exact underlying mechanism is not well understood. In previous studies, miRNA could mediate the post-transcriptional regulation of Myog. miR-186 inhibited the differentiation of C2C12 and primary muscle cells by targeting the Myog 3′-UTR (Antoniou et al., 2014). The expression levels of miR-2400 were downregulated during muscle-derived satellite cells (MDSCs) differentiation; miR-2400 could promote muscle-derived satellite cells proliferation through targeting Myog 3′-UTR in bovine (Zhang et al., 2015). miR-2425-5p promoted MDSCs proliferation and inhibited the MDSCs differentiation by binding to Myog 3′-UTR in bovine (Tong et al., 2017). In a recent study, lncRNA SYISL recruited the enhancer of zeste homolog2 protein, the core component of polycomb repressive complex 2, to the Myog promoter, leading to H3K27 trimethylation and epigenetic silencing of Myog to inhibit myogenic differentiation (Jin et al., 2018). In addition, the lncRNA MUNC from a heterologous promoter enhanced Myog mRNA in the trans, but not cognate protein (Mueller et al., 2015). To the best of our knowledge, this is the first study in which lnc-GD2H was shown to promote the mRNA and protein levels of Myog *via* transcriptional regulation to facilitate myoblast differentiation. In summary, lncRNAs play important roles in the regulatory network of Myog, and several additional mechanisms involving them may exist that should be further investigated.

c-Myc is a multifunctional transcription factor that regulates various processes. c-Myc regulates the lncRNA NEAT1 to promote B-cell proliferation and lymphomagenesis in diffuse large B-cell lymphomas (Qian et al., 2020). Additionally, c-Myc targets the antisense RNA 1 of the lncRNA RHPN1, which promotes breast cancer cell proliferation (Zhu et al., 2019). c-Myc also plays a significant role in muscle development. c-Myc mutation in homozygotes is lethal during gestation; the embryos were generally smaller and retarded in development compared with their littermates (Davis et al., 1993). Furthermore, c-Myc is a significant regulator of the cell cycle, and its knockdown or knockout severely influences cell proliferation (Bouchard et al., 1999; Perez Roger et al., 1999). In mouse embryo stem cells, c-Myc inhibited the expression of differentiation marker genes and affected stem cell differentiation by inducing miRNA expression (Lin et al., 2009). In the present study, c-Myc regulated lnc-GD2H transcription to promote myoblast proliferation. This was consistent with previous research showing that long intergenic non-coding RNAs (lincRNAs) can be directly regulated by c-Myc and the c-Myc-regulated targets were important for chicken skeletal muscle development (Luo et al., 2019). It had also been reported that the expression of c-Myc was downregulated after the beginning of differentiation in C2C12 myoblasts (Yeilding et al., 1998); whereas, lnc-GD2H was significantly upregulated from 0 to 96 h in the differentiation medium of the present study. Therefore, we speculated that other transcription factors should regulate lnc-GD2H during myoblast differentiation, which needs further study.

The present study has potential limitations. Although the results showed that the effects of lnc-GD2H on C2C12 cell proliferation *in vitro* depended on c-Myc, a feedback loop formed between lnc-GD2H and c-Myc, and c-Myc could regulate lnc-GD2H transcription to promote myoblast proliferation, the mechanism by which lnc-GD2H regulates c-Myc remains unclear. Reportedly, the development-promoting effects of c-Myc are mediated *via* a CDK2-, CDK4-, or CDK6-dependent pathway (Zhong et al., 2006; Bouillez et al., 2016; Kawasaki et al., 2016; Ouyang et al., 2016; Liao et al., 2017; Zhang et al., 2019). In the present study, lnc-GD2H regulated CDK2, CDK4, and CDK6, and whether lnc-GD2H regulates c-Myc *via* a CDK2-, CDK4-, or CDK6-dependent pathway warrants further investigation. Proliferation and differentiation were two vital stages of skeletal muscle development; however, the switch mechanism of the two processes was also significant. lnc-GD2H was involved in regulating of the two stages, whether it mediated the switch of these two stages remained to be further studied.

## Conclusion

In summary, the study results showed that lnc-GD2H expression was enhanced during myoblast proliferation and differentiation. Moreover, lnc-GD2H facilitated the expression of proliferating marker genes and formed a feedback loop with c-Myc during myoblast proliferation. In differentiating myoblasts, lnc-GD2H interacted with NACA to alleviate the inhibitory effect of NACA on Myog, thereby promoting Myog expression to facilitate differentiation. These results aid in understanding the mechanisms by which lncRNAs govern myoblast proliferation and differentiation. In addition, this information provides evidence for the function of lncRNA in muscle regeneration and is useful for the development of novel therapeutic targets for muscle disorders.

## Data Availability Statement

The datasets presented in this study can be found in online repositories. The names of the repository/repositories and accession number(s) can be found below: ProteomeXchange Consortium via the PRIDE partner repository with the dataset identifier PXD025292 (Project Webpage: http://www.ebi.ac.uk/pride/archive/projects/PXD025292).

## Ethics Statement

The animal study was reviewed and approved by Ethics Committee of Guangdong Second Provincial General Hospital.

## Author Contributions

RC, CL, and TJ conceived and designed the experiments. RC, SL, YS, HS, and SZ performed the experiments *in vitro* and analyzed the data. RC, YS, and SZ performed the experiments *in vivo* and analyzed the data. RC and SL wrote the manuscript. All authors read and approved the final manuscript.

## Conflict of Interest

The authors declare that the research was conducted in the absence of any commercial or financial relationships that could be construed as a potential conflict of interest.

## Publisher’s Note

All claims expressed in this article are solely those of the authors and do not necessarily represent those of their affiliated organizations, or those of the publisher, the editors and the reviewers. Any product that may be evaluated in this article, or claim that may be made by its manufacturer, is not guaranteed or endorsed by the publisher.

## Footnotes

1
http://jaspar.genereg.net/

2
https://www.uniprot.org/

3
http://pridb.gdcb.iastate.edu/RPISeq
