# Supplementary material for: Lnc-GD2H Promotes Proliferation by Forming a Feedback Loop With c-Myc and Enhances Differentiation Through Interacting With NACA to Upregulate Myog in C2C12 Myoblasts
Source: Front Cell Dev Biol. 2021 Aug 18;9:671857. doi: 10.3389/fcell.2021.671857 (PMC8416608; doi:10.3389/fcell.2021.671857)
Supplement: Supplementary file 1 [file Data_Sheet_1.docx]

**Supplementary**


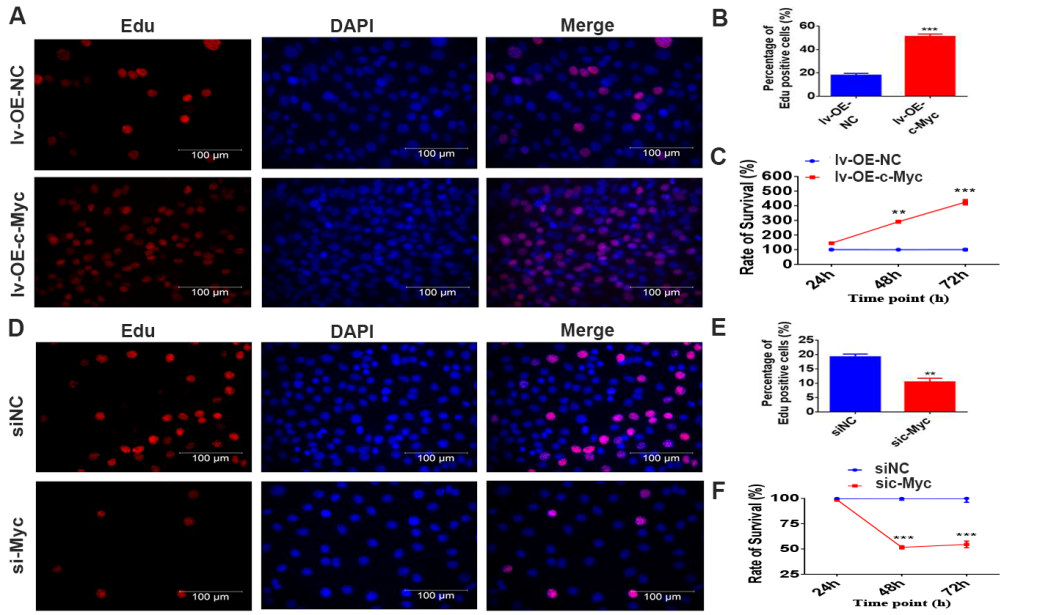


**Supplementary Figure 1.** Impacts of c-Myc on proliferation of C2C12.**(A)** Cell proliferation was investigated using theEdU assay in c-Myc-overexpressingcells. **(B)** Percentage of EdU-positive cells inc-Myc-overexpressingcells. **(C)** Cell survival was determined using the CCK-8 assayin c-Myc-overexpressingcells. **(D)**EdU assay results for c-Myc-knockdown cells. **(E)** Percentage of EdU-positive cells in c-Myc-knockdown cells. **(F)** Cell survival was determined using the CCK-8 assay in c-Myc-knockdown cells.


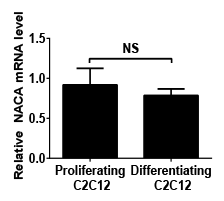


**Supplementary Figure 2.** The expression level of NACA in proliferating C2C12 and differentiating C2C12.

**Supplementary Table 1.**Sequences of the mRNA and lncRNA primers

| Gene | Forward primer (5'-3') | Reverse primer (5'-3') |
| --- | --- | --- |
| 18s | GTAACCCGTTGAACCCCATT | CCATCCAATCGGTAGTAGCG |
| lnc-GD2H | GCTAGCATAGCCACCCTGTC | CACAGCACTGAATGCCATCG |
| Myog | GGCAATGCACTGGAGTTCG | AGCCGCGAGCAAATGATC |
| c-Myc | CCCTATTTCATCTGCGACGAG | GAGAAGGACGTAGCGACCG |
| CDK2 | GCGACCTCCTCCCAATATCG | GTCTGATCTCTTTCCCCAACTCT |
| CDK4 | ATGGCTGCCACTCGATATGAA | TCCTCCATTAGGAACTCTCACAC |
| CDK6 | GGCGTACCCACAGAAACCATA | AGGTAAGGGCCATCTGAAAACT |
| Mef2a | CAGGTGGTGGCAGTCTTGG | TGCTTATCCTTTGGGCATTCAA |
| Mef2c | ATCCCGATGCAGACGATTCAG | AACAGCACACAATCTTTGCCT |
| NACA | TCGCATACGGTCTTGGCG | GTGGCGCGGTCTAAATAAGG |
